# QSAR modeling and docking analysis of D2 receptor with known olanzapine derivatives

**DOI:** 10.6026/97320630016666

**Published:** 2020-09-30

**Authors:** Kiran Bhargava, Prahlad Kishore Seth, Kamlesh Kumar Pant, Rakesh Kumar Dixit, Vivek Agarwal, Kamal Kumar Sawlanid, Rajendra Nath

**Affiliations:** 1 King George's Medical University Erstwhile CSMMU, Lucknow, UP, India; 2 Biotech Park, Lucknow, UP, India

**Keywords:** Schizophrenia, Olanzapine derivatives, D2 receptor, QSAR, Antipsychotic agents

## Abstract

Dopamine (D2) receptors are known drug targets for various antipsychotics used in Schizophrenia. Therefore, it is of interest to analyze the binding features of D2 receptors with known olanzapine derivatives for further consideration using molecular docking and QSAR analysis. A 2D QSAR model was built using energy-based descriptors generated by docking as independent variable and known Ki value of Olanzapine derivatives with D2 Receptor as dependent variable. QSAR model provided coefficient of determination of r2 of 0.7 in multiple linear regression analysis. The predictive performance of QSAR model was assessed using different cross-validation procedures. Thus, data shows that a ligand-receptor binding interaction for D2 Receptor using a QSAR model is promising approach to design novel and potent inhibitors of D2 Receptor.

## Background

In today world, mental disorders have become highly prevalent because of numerous reasons like urbanization, ambitious lifestyle and stressful environment [1]. A mental disorder is health conditions involving changes a person's normal thinking, feelings, mood, behaviour and that causes difficulty in person functioning. The majority of the people don't know that they are experiencing the side effects of mental issue on the grounds that in the underlying stage the side effects is mild and later on it become a serious mental disorder. Schizophrenia is a mental disorder characterized by distortions in thinking, perception, feels, behaviour and sense of self. People with schizophrenia often have problems doing well in society, at work, at school, and seeing someone [2]. They may feel alarmed and pulled back, and could seem to have put some distance with realty. Schizophrenia can't be cured but can be controlled with proper treatment. People with schizophrenia are 2-3 times more likely to die at a younger age than the normal one [3].

Worldwide, Schizophrenia affecting 20 million people [2] and is described in terms of positive and negative symptoms. Characteristics of schizophrenia typically include hallucinations or delusions as positive symptoms and negative symptoms as poverty of speech and impairments in cognition. Schizophrenia etiology shows that numerous variables are included, in particular genetic factors [4,5] changes in chemical transmission [6], obstetrical complications [7] and viral Infections [8]. The prevalence of schizophrenia approaches 1% worldwide. The incidence is about 1.5 per 10,000 people [9].

The patho mechanism of schizophrenia is not fully understood and current antipsychotics had side effects. Second line antipsychotics treat mainly positive symptoms but negative and cognitive symptoms remain untreated [10]. D2 receptors [11] are widely expressed in the human brain and are very important from a pharmacological point of view, as they constitute the target site of many centrally acting drugs. Therefore, it is of interest to analyze the binding features of D2 receptors with known olanzapine derivatives for further consideration using molecular docking and QSAR analysis.

## Methodology

### Receptor and ligands preparation:

The 3D model structure of D2 receptor from Homo sapiens was retrieved from our previous published paper [12] for docking studies. Olanzapine and its derivatives with known Ki were obtained from literature [13]. Derivatives were building using PubChem Sketcher V2.4 [14] and after converted in 3D structures CORINA tool. All the ligands were subjected to energy minimization using the HyperChem software [15].

### Molecular docking:

Molecular docking is convenient tool utilized in the drug discovery process to investigate the binding compatibility of ligands to receptor [16]. Olanzapine and its 11 derivatives screened from literature against D2 Receptor structure was done by molecular docking program AutoDock 4.2 [17]. The Lamarckian genetic algorithm implemented in Autodock was used for docking process.

### 2D QSAR:

A QSAR based model was developed using inhibitory activities of Olanzapine and its derivatives with D2 receptor represented as pKi values and six types of energy value such as Binding Energy (BE), Intermolecular Energy (IME), Internal Energy (IE), Torsional Energy (TorE), vdW + Hbond + desolv Energy (VdwE) and electrostatic energy (EE) as descriptors. Several cross-validation systems were adopted to evaluate the predictive performance of the QSAR model.

## Results and Discussion:

**Figure 1:**
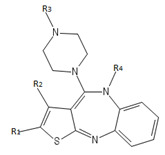
Olanzapine derivatives

**Figure 2:**
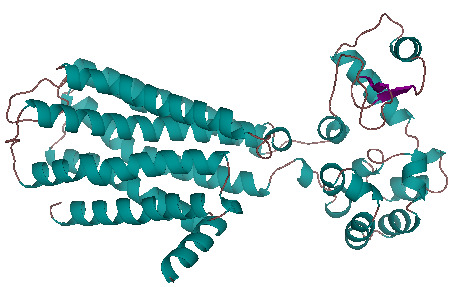
Model structure of D2 receptor

Olanzapine and its derivatives based on R1, R2, R3 and R4 groups in Figure 1 at different positions was shown in Table 1 (see PDF). Molecular docking study was carried out between Olanzapine derivatives and D2 receptor structure and best autodock score was used as criteria to interpret the best conformation among the 30 conformations, generated by AutoDock 4.2 program. Model structure of D2 receptor was shown in Figure 2. All the derivatives were found to inhibit the receptor by occupying the active sites of D2 receptor. For target protein, binding affinity values for all the compounds range from -2.99 to -7.43 kcal/mol as reported in Table 2 (see PDF).

**Figure 3:**
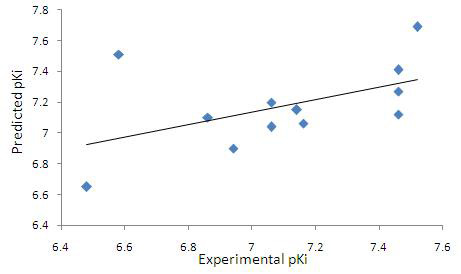
Relationship between experimental (x-axis) and predicted (y-axis) pKi values with an r2 value 0.70283 is shown in a QSAR model developed using multiple linear regression analysis.

QSAR was performed to investigate the structure-activity relationship of Olanzapine derivatives as potent antipsychotics agents. A correlation coefficient (r2) of 0.70283 was obtained for Olanzapine derivatives as shown below in equation 1. Experimental and predicted activities for Olanzapine derivatives were shown in Table 1 (see PDF). The low residual value between experimental and predicted activity indicates that the model is of high predictability. Relationship between experimental and predicted pKi values of Olanzapine derivatives was shown in Figure 3.

The key accomplishment of the Quantitative structure-movement relationship (QSAR) strategy is the likelihood to anticipate the properties of new compound without the need to synthesize and test them. This strategy is comprehensively used for the prediction of physicochemical properties in the compound in pharmaceutical industry [18]. A lot of research works reported treatment of schizophrenia based on dopamine (D2) receptor as drug target. Such as de Haan et al. reported a level of D2 receptor occupancy between 60% and 70% is optimal for subjective experience of patients with recent-onset schizophrenia. A considerable interindividual variation in occupancy was resulted at fixed low level dose of olanzapine and haloperidol compounds [19]. Lavalaye et al. assessed striatal dopamine D2 receptor occupancy by olanzapine and risperidone in young patients with first episode schizophrenia and found that both drugs induce a high occupancy, depending on dose as well as group formation [20]. A selective antagonist (JNJ-37822681) of D2 receptor treatment in patients of schizophrenia was connected with more favorable results on weight and metabolic adverse effects compare to olanzapine [21].

## Conclusion

A QSAR model was developed using Ki value of known olanzapine derivatives with the D2 receptor as dependent variable. The six energy based descriptors namely binding energy, intermolecular energy, internal energy, torsion energy, vdW + H bond + desolv energy and electrostatic energy as independent variable. The coefficient of determination r2 is 0.7. Thus, we document the QSAR modelling and docking analysis of D2 receptor with known olanzapine derivatives for further consideration.

## References

[1] Milind P (2013). Int Res J Pharm.

[2] GBD 2017 Disease and Injury Incidence and Prevalence Collaborators (2018). The Lancet.

[3] Laursen TM (2014). Review of Clinical Psychology.

[4] Levy DL (2010). J Neurolinguistics.

[5] Alaerts M (2009). Hum Mutat.

[6] Lipska BK (2004). J Psychiatry Neurosci.

[7] Ho BC (2010). Neuroimage.

[8] Brown AS (2010). Am J Psychiatry.

[9] McGrath J (2008). Epidemiol Rev.

[10] Stepnicki P (2018). Molecules.

[11] Lau CL (2013). Rev Neurosci.

[12] Bhargava K (2014). Bioinformation.

[13] Avram S (2011). J Serb Chem Soc.

[14] https://pubchemncbinlmnihgov/edit2/indexhtml.

[15] HyperChem Group (2020). HyperChem (TM) Release 75 Manual.

[16] Hawkins VAG (2007). Journal of Medicinal Chemistry.

[17] Morris GM (2009). J Computational Chemistry.

[18] Wong KY (2004). Journal of Biomedical Science.

[19] de Haan L (2003). Am J Psychiatry.

[20] Lavalaye J (1999). Psychiatry Res.

[21] Daly EJ (2013). Ann Clin Psychiatry.

